# Interstitial cells of Cajal plasticity rather than regeneration restores slow-wave activity and enteric neurotransmission upon acute damage

**DOI:** 10.1186/2050-6511-14-S1-P34

**Published:** 2013-08-29

**Authors:** Sabine Klein, Barbara Seidler, Anna Kettenberger, Andrei Sibaev, Robert Feil, Franz Hofmann, Jean-Marie Vanderwinden, Hans-Dieter Allescher, Michael Schemann, Martin A Storr, Roland M Schmid, Günter Schneider, Dieter Saur

**Affiliations:** 1 Department of Internal Medicine 2, Klinikum rechts der Isar, Technische Universität München, 81675 München, Germany; 2 Department of Internal Medicine 2, Ludwig-Maximilians-Universität München, 81377 München, Germany; 3 Interfaculty Institute of Biochemistry, University of Tübingen, 72076 Tübingen, Germany; 4 Institut für Pharmakologie und Toxikilogie, Klinikum rechts der Isar, Technische Universität München, 80802 München, Germany; 5 Université Libre de Bruxelles, ULB Neuroscience Institute, Laboratory of Neurophysiology, Faculty of Medicine, B-1070 Brussels, Belgium; 6 Zentrum für Innere Medizin, Klinikum Garmisch-Patenkirchen, 82467 Garmisch-Patenkirchen, Germany; 7 Lehrstuhl für Humanbiologie, Technische Universität München, 85350 Freising-Weihenstephan, Germany

## Background

The enteric nervous system contains excitatory and inhibitory neurons which control contraction and relaxation of smooth muscle cells and gastrointestinal (GI) motor activity. Nitric oxide (NO) plays an important role as a non-adrenergic non-cholinergic inhibitory neurotransmitter in the enteric nervous system, which activates the NO-GC/cGMP/PKG signalling pathway and thus relaxation of the smooth musclulature in the GI tract. Interstitial cells of Cajal (ICC) act as pacemaker cells in the GI tract by generating slow waves of depolarisation to induce rhythmic smooth muscle contractions. In addition, our previous work established a surprising role of ICCs in excitatory and inhibitory nitrergic neurotransmission. The aim of the present study was to investigate molecular and cellular mechanisms, which mediate regeneration of intestinal slow-waves and inhibitory nitrergic neurotransmission upon acute damage of the ICC network.

## Materials and methods

To evaluate the role of ICC in excitatory and NO-dependent inhibitory neurotransmission after acute damage, we generated a *c-Kit^CreERT2^* knock-in allele at the endogenous *c-Kit* locus. This tamoxifen inducible mouse model enables genetic manipulation and depletion of ICC as well as regeneration studies at defined time points during development and in adults *in vivo*. To investigate the role of ICC in transducing the nitrergic inhibitory signal, we deleted *cGMP-dependent protein kinase I* (*Prkg1*), the central mediator of the non-adrenergic, non-cholinergic neurotransmission in ICC using floxed *Prkg1* animals. Furthermore we crossed *c-Kit^CreERT2/^*^+^ mice with conditional *LSL-R26^DTA/^*^+^ animals, which carry a latent diphtheria toxin A (DTA) expression cassette to deplete the ICC network. Using these models, we subsequently investigated molecular and cellular mechanisms which mediate regeneration of slow-waves and GI motility over time.

## Results

Deletion of *Prkg1* in ~40% of all ICC abolished specifically the NO-dependent component of the inhibitory junction potential in colonic circular smooth muscle cells. This resulted in a significantly disturbed GI motility with a profound increase in total GI transit time, as seen in animals with a disruption of the ICC network due to expression of DTA. Interestingly, GI motility, slow-wave activity and enteric neurotransmission recovered completely within 5 weeks. However, we found no overt recovery of ICC cell number, reexpression of *Prkg1* or proliferation of precursor cells.

## Conclusion

Our results suggest that adaptive mechanisms of the remaining ICC restore pacemaker activity and enteric neurotransmission. Therefore, we provide first *in vivo* genetic evidence for a surprising plasticity of ICC which restores normal gut function after damage of the ICC network.

